# Reforestation-induced changes of landscape composition and configuration modulate freshwater supply and flooding risk of tropical watersheds

**DOI:** 10.1371/journal.pone.0181315

**Published:** 2017-07-14

**Authors:** Qiong Gao, Mei Yu

**Affiliations:** Department of Environmental Sciences, University of Puerto Rico - Rio Piedras, San Juan, Puerto Rico, United States of America; Chinese Academy of Forestry, CHINA

## Abstract

Impact of changes in land cover and land use on hydrological service of tropical watersheds is one of the focal research tropics in both hydrology and Land Cover Land Use Changes (LCLUC). Land fragmentation is an important feature of LCLUC, however, its impact on hydrological service of tropical watershed is unclear despite a few theoretical frameworks. In this paper, we described a simulation study of eight tropical watersheds in Puerto Rico using the Soil Water Assessment Tool. Annual average stream discharge was derived according to the simulations with the land cover maps in 1977, 1991, and 2000. Annual big stream discharge with risks of flooding and severe soil erosion was computed as the sum of daily discharge greater than 95^th^ percentile. The impacts of changes in land cover and fragmentation represented by perimeter-to-area ratio of land patches on annual average and big discharges were further analyzed by means of the linear mixed-effects model. Most mountainous watersheds were characterized by reforestation in 1977–1991 but slight deforestation in 1991–2000. Forest perimeter-to-area ratio was significantly correlated with covers of forest (correlation coefficient of -0.97), pasture (0.94), and urban (0.95). Thus forest fragmentation was reduced by reforestation but increased by deforestation. The annual average and big discharges were significantly reduced by forest cover and forest perimeter-to-area ratio. The enhanced edge effect by forest fragmentation may have incurred more effective interception of the subsurface flow by forest root system, and promoted forest transpiration, thus reduced stream flows. Land cover change plays more important roles in regulating the big discharges than altering the annual average discharges. Due to the negative correlation between forest cover and fragmentation, the decreased forest fragmentation accompanied with reforestation offsets the impact of reforestation on lessening freshwater supply and flooding risk.

## Introduction

Tropical and subtropical watersheds host the ecosystems with great carbon storage and carbon sequestration ability to offset the Green House Gas emission [1], and provide hydrological services for dense population [2]. Recent projection of global warming and drying casts many concerns on the adaptation of tropical ecosystems and the sustainability of ecosystem services [3, 4].

The drying climate in the Caribbean region has been reported for the past [5]. And the drying trend is also projected for this century as one of the strongest in the globe [6] characterized by 0.7–4°C increase in temperature, 25–50% reduction in rainfall [7], and increased chaotic extremes such as droughts and storms. The drying trend in the Caribbean threatens the tropical vegetation and the hydrological service to the dense population [8]. Small islands in general have little buffering capacity, moreover, the economic crisis in island countries such as Puerto Rico disabled their capability to maintain the capacity of reservoirs. Consequently, a moderate drought can lead to insufficient water supply [9].

In addition to the drying climate, changes in land cover and land use (LCLUC) may also modulate the hydrological service [10]. The Caribbean region has been experiencing urbanization, urban sprawl, and various extents of reforestation and deforestation [11, 12]. For example, Puerto Rico’s reforestation followed the industrialization of the island in late 1940s and the forest cover increased from 6% to more than 46% in 2000 [13, 14]. Forest cover in Dominic Republican increased from about 12–15% to about 30% in the similar period (http://www.diccionariomedioambiente.org/). Deforestation has also been found in the Caribbean, especially in Haiti where charcoal is the main household energy source [12, 15].

Forest transpires water to atmosphere, recycles part of the transpired water as rainfall [16], and intercepts great amount of water by its large leaf area. Except the wet and the cloud forests, the net effect of forest on hydrological service is negative [17], and forest in general transpires more water than other vegetation types. On the other hand, extensive forest root system facilitates the recharge to deep soils, thus enhances ecosystem water storage capacity to prevent disastrous flooding or severe erosion [18, 19]. Since the Caribbean is dominated by the moist and the dry forests [12], reforestation in this region tends to reduce total freshwater supply due to increased forest transpiration. In the mountainous islands reforestation also increases ecosystem buffer for extreme rainfall and thus smoothens stream flow to reduce the chance of flooding and severe erosion such as landslides [19]. However, the strength of these effects for the Caribbean still lacks cohesive conclusion.

Changes in land use in general alter landscape configuration. The deforestation or forest regrowth at landscape scale is limited not only by physical environmental conditions such as elevation, slope, soil, and parent materials [20], but also more importantly by the human activities such as urbanization, desire for agriculture due to the growing population, and other socioeconomic demand [21, 22]. Consequently, landscape fragmentation has been found as a main trend of LCLUC in many places of the world [23, 24].

Land fragmentation impacts various aspects of ecosystem functions and services. A common assumption is that fragmentation reduced the supply of ecosystem services. However, the effect of fragmentation on the service delivery can be positive or negative [25]. In terms of hydrological service, fragmentation brings more edges or boundaries which expose forest to more radiation, growth, and transpiration, but less stream discharge [26, 27]. Enhanced edges may also promote effective interception of the subsurface flows by the deep root system to facilitate more evapotranspiration. Fragmentation with diverse vegetation allows efficient precipitation redistribution and reuse of runoff so as to give less discharge in streams in semiarid and arid systems [28–30]. On the other hand, forest fragmentation in the tropical area may leave passages for overland or subsurface flow generated by other vegetation types from upper slope [31], a situation similar to the effects of fragmented Tigger Bush [32]. The net effect of changes in landscape configuration is largely unknown for regions with diverse reforestation-deforestation intensities.

This study intends to explore how changes in landscape composition and configuration impact the hydrological service of tropical watersheds in the Caribbean region. We attempt to test two hypotheses: (1) Freshwater service of tropical watersheds is affected by landscape composition and fragmentation, in additional to the main control of rainfall; and (2) Big stream flow with flooding and erosion risks, defined by daily discharge greater than 95^th^ percentile, is more affected by variation of landscape composition and fragmentation than the average stream discharge.

We used the Soil Water Assessment Tool (SWAT) [33–35] to simulate the discharges of eight tropical watersheds in Puerto Rico using three land cover/use maps from 1977 to 2000. The modeling results were checked with the USGS long-term gauged stream discharges. The average discharge and the big flow were further analyzed to derive relationship between these variables and the variables of landscape composition and configuration.

## Methods

### Watersheds

**Fig 1:**
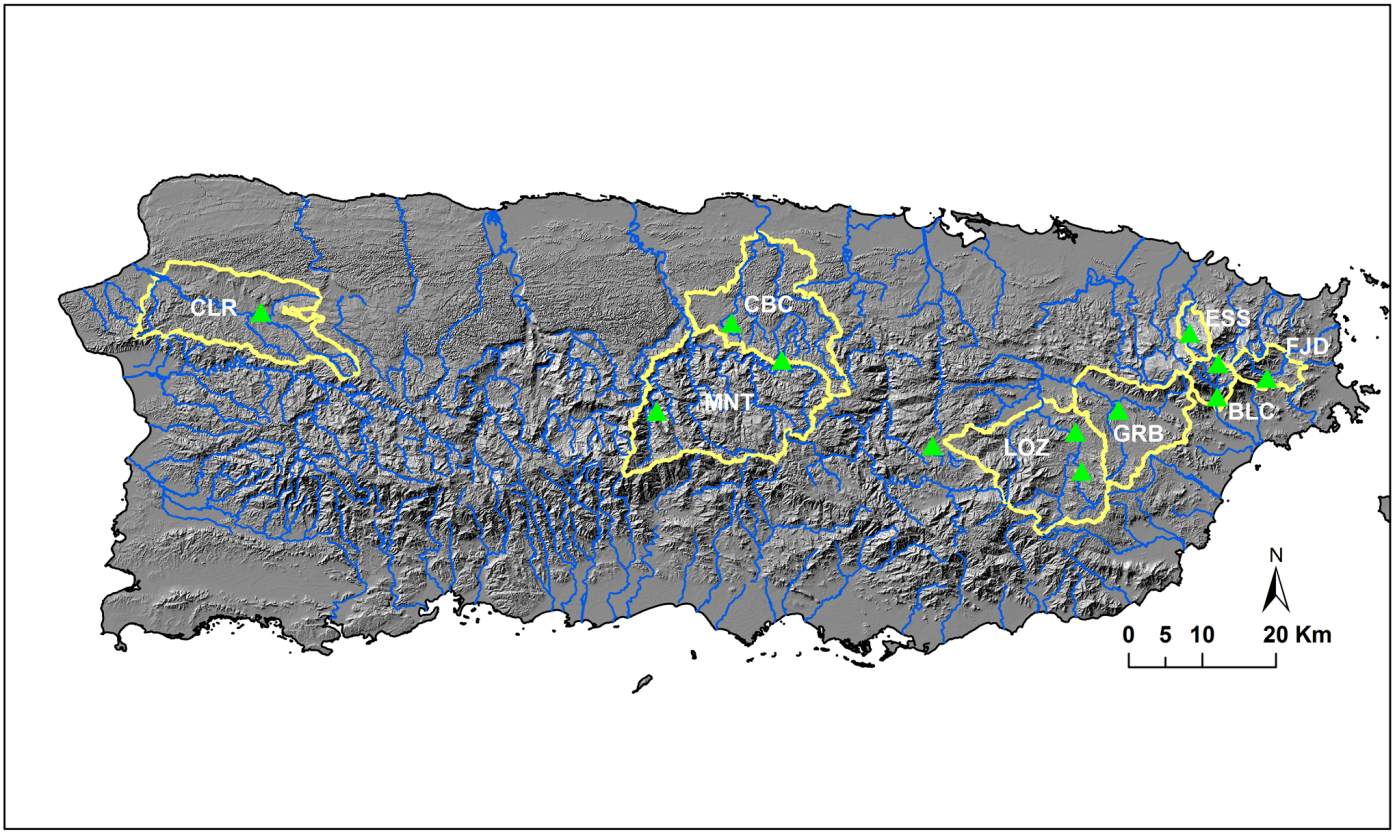
Watersheds in this study. FJD, Rio Fajardo, ESS, Rio Espiritu Santo, BLC, Rio Blanco, GRB, Rio Gurabo, LOZ, Rio Grande de Loiza, CBC, Rio Cibuco, MNT, Rio Grande de Manati, and CLR, Rio Culebrinas. Green points represent the meteorological stations used in the simulation. The background topography information is for illustration only (Data publicly accessible from https://lta.cr.usgs.gov/SRTM1Arc).

We chose eight tropical watersheds in Puerto Rico based on the following criteria: (1) the watersheds should have USGS gauged discharge observations lasting for at least the past 20 years until 2010; (2) due to the lack of long-term water use data, watersheds with reservoirs are not considered except the Rio Grande de Manati watershed; and (3) the watersheds should have at least one meteorological station within or nearby with 80% or more complete daily rainfall and temperature records. The Rio Grande de Manati is an important watershed because the river originates from the highest peak in Puerto Rico and delivers large amount of freshwater to the metropolitan areas. We intended to do a water discharge simulation for this watershed despite two reservoirs, i.e., El Guineo and Matrullas, within it. The names, spatial locations, USGS discharge gauge codes, and brief description of selected watersheds were illustrated in Fig 1 and Table 1. These watersheds are mostly the mountainous upper reaches of large watersheds in Puerto Rico. Three watersheds, i.e., Rio Espiritu Santo (ESS), Rio Blanco (BLC), and Rio Fajardo (FJD), originate from the eastern Luquillo Mountains with great rainfall and high forest cover. Rio Grande de Loiza near Caguas (LOZ) and Rio Gurabo (GRB) are the upper parts of the Rio Grande Loiza watershed which spans from the mountains to the northern coastal wetlands. Rio Grande de Manati at Ciales (MNT) and Rio Cibuco (CBC) are located in the Karst area with unknown leaking to the underground streams. Rio Culebrinas (CLR) is located in the northwestern corner (Fig 1).

**Table 1:** Description of the eight watersheds in Puerto Rico.

Watershed	USGS code	Average elevation a.s.l. (m)	Area (km^2^)
Rio Fajado (FJD)	50071000	269.6	37.9
Rio Espiritu Santo (ESS)	50063800	442.2	22.4
Rio Blanco (BLC)	50076000	525.9	31.5
Rio Gurabo (GRB)	50057000	169.2	153.6
Rio Grande de Loiza (LOZ)	50055000	265.8	229.6
Rio Cibuco (CBC)	50039500	183.5	224.2
Rio Grande de Manati (MNT)	50035000	578	326.9
Rio Culebrinas (CLR)	50148890	144.3	244.5

### Data preparation

Hydrological simulation was carried out by means of the Soil Water Assessment Tools (SWAT) (http://swat.tamu.edu) which needs geomorphology, soil, and land use information as inputs. Daily rainfall and temperature from the meteorological stations within or nearby the watershed were utilized to drive the model. We preprocessed the DEM at 10m resolution (www.gis.pr.gov), the soil map and soil properties from the Soil Survey Geographic database (SSURGO) [36], and the land cover/use maps in 1977, 1991, and 2000 [37]. The land cover/use maps of 1991 and 2000 were digital-classified based on the Landsat images, but the map of 1977 was visually interpreted from the aerial photos [37] and has much lower resolution than the Landsat derived maps.

Daily climate records were mostly from the NOAA stations for all the watersheds except the Rio Espiritu Santo, for which the long-term rainfall records from the El Verde field station of the University of Puerto Rico were applied. Missing temperature data was interpolated based on the long-term averages, but missing rainfall was scaled from that of the nearest station. The ‘borrowed’ rainfall assumed daily synchronization among stations thus may incur error in the simulation.

### The SWAT model and calibration

SWAT simulates the hydrological processes of a watershed together with the vegetation growth [33–35, 38, 39]. The hydrological components in the model include rainfall, soil water, shallow groundwater, evapotranspiration, and surface/subsurface flows. The modeled stream discharge is usually validated against the gauged discharge at the outlet of watershed. Nutrient dynamics as well as sediment/chemical transportation in the water and the soil are also considered.

SWAT is neither a lumped nor a pixel-based model. The basic simulation unit is the hydrological response unit (HRU) defined by the overlay of sub-basins, soil types, land cover/use, and slope categories. Surface and subsurface flows are routed to streams and rivers in the watershed. Parameters of soil and vegetation are connected to the built-in SWAT database and can be manually adjusted. The DEM, land cover/use, and soil data are processed by the ArcSWAT module during the watershed delineation and the HRU definition. We set 200 hectare as the minimum sub-basin area to limit the details in the watershed delineation except BLC and ESS, for which 20 ha was used. We used three slope ranges with equal area as slope categories, and 15% as the minimum sub-basin area in the HRU definition.

SWAT was calibrated for each watershed manually for the discharge gauged during 1996–2005 and the land cover map of 2000. The curve number (cn2) was adjusted for appropriate runoff ratio. Coefficient of plant uptake (EPCO), soil evaporation (ESCO) from deep soil layer, and available soil water content (SOL_AWC) were adjusted for evapotranspiration. Saturated soil hydraulic conductivity (SOL_K), threshold water depth in the shallow aquifer required for return flow (GWQMN), coefficient controlling upward movement from shallow aquifer to overlaying unsaturated zone (QW_REVAP), and threshold depth of shallow aquifer allowing REVAP or percolation to deep aquifer (REVAPMN) were adjusted for appropriate lateral flow, evaporation from the shallow aquifer, and percolation to the deep aquifer (SWAT 2012 Input / Output document). All the parameters were adjusted within 20% of the nominal values.

### Simulation using observed meteorological records from the 1970s to the end of 2013

For each watershed, we did simulation based on each of the three land cover/use maps with the same set of climate and soil data. The outputs included monthly and daily stream discharges at the outlets of the watershed and the sub-basins, as well as rainfall and evapotranspiration. The monthly discharge simulated with the land cover/use of 2000 was compared to the USGS gauged discharge. The quality of simulation was assessed by calculating Pearson’s correlation coefficient between the gauged and the simulated discharges from 1996 to 2013, and root mean square of error. The 95^th^ percentile of daily discharge was calculated from the simulated daily discharge with the land cover/use in 1977. Daily discharges greater than the percentile were defined as big stream flows which tend to cause flooding and severe erosion, and then cumulated for each year.

During the post simulation analysis, we grouped the land cover/use types into four categories, i.e., urban, agriculture, grassland/pasture, and forest, and calculated the land cover fraction and the average perimeter-to-area ratio of each category. Variations in land cover fraction and perimeter-to-area ratio represent changes in composition and configuration, respectively. We divided the long-term average and big discharges for each watershed by watershed area to give flow per watershed area. The average annual and big discharges per watershed area were then regressed on rainfall, land cover fraction, and perimeter-to-area ratio using the linear mixed-effects model with watershed as a grouping variable. Linear mixed-effects model is intended to analyze structured data with a fixed effect component and a random effect component [40]. The fixed effect component gives the parameters for the entire population, and the random effect component is in general for individual experimental unit. In this study, we intended to derive the relationship between hydrological service and changes in land cover in the fixed effect component. We also wanted to fit a random intercept for each watershed to account for difference in factors other than changes in climate and land cover among watersheds. Because the resolution of the land cover/use map in 1977 is much coarser than that in 1991 and 2000, and calculation of fragmentation index is highly sensitive to the resolution [20], the application of linear mixed-effects model was carried out in two steps, i.e., the regressions of simulations using maps of 1977 and 1991 without, but those of 1991 and 2000 with, the perimeter-to-area ratios as explanatory variables. The free statistical software R [41] was used throughout the data preparation and the post simulation analyses. Functions of the LME in NLME package and the LMER in LME4 package were used. Backward stepwise selection of the independent variables was done manually to minimize the Akaike Information Criterion (AIC) for the fixed-effect component of the model.

## Results

### Comparison of the simulated watershed discharges to the USGS observations

**Fig 2:**
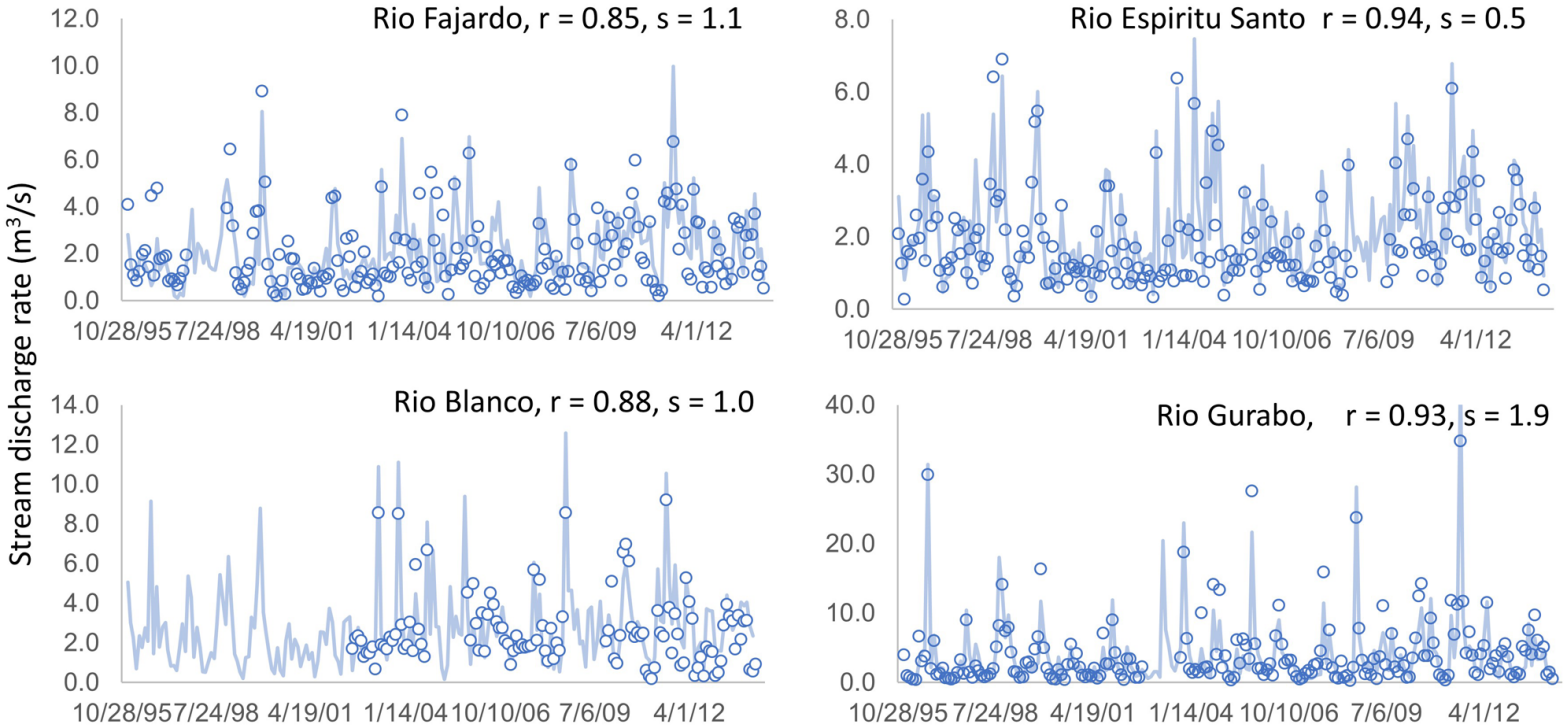
Simulated monthly mean discharge with the land cover map of 2000 versus USGS gauged data for FJD, ESS, BLC, and GRB. All the correlation coefficients have *p*-values less than 0.01. *r* is the Pearson’s correlation coefficients, *s* is the root mean square of the error.

**Fig 3:**
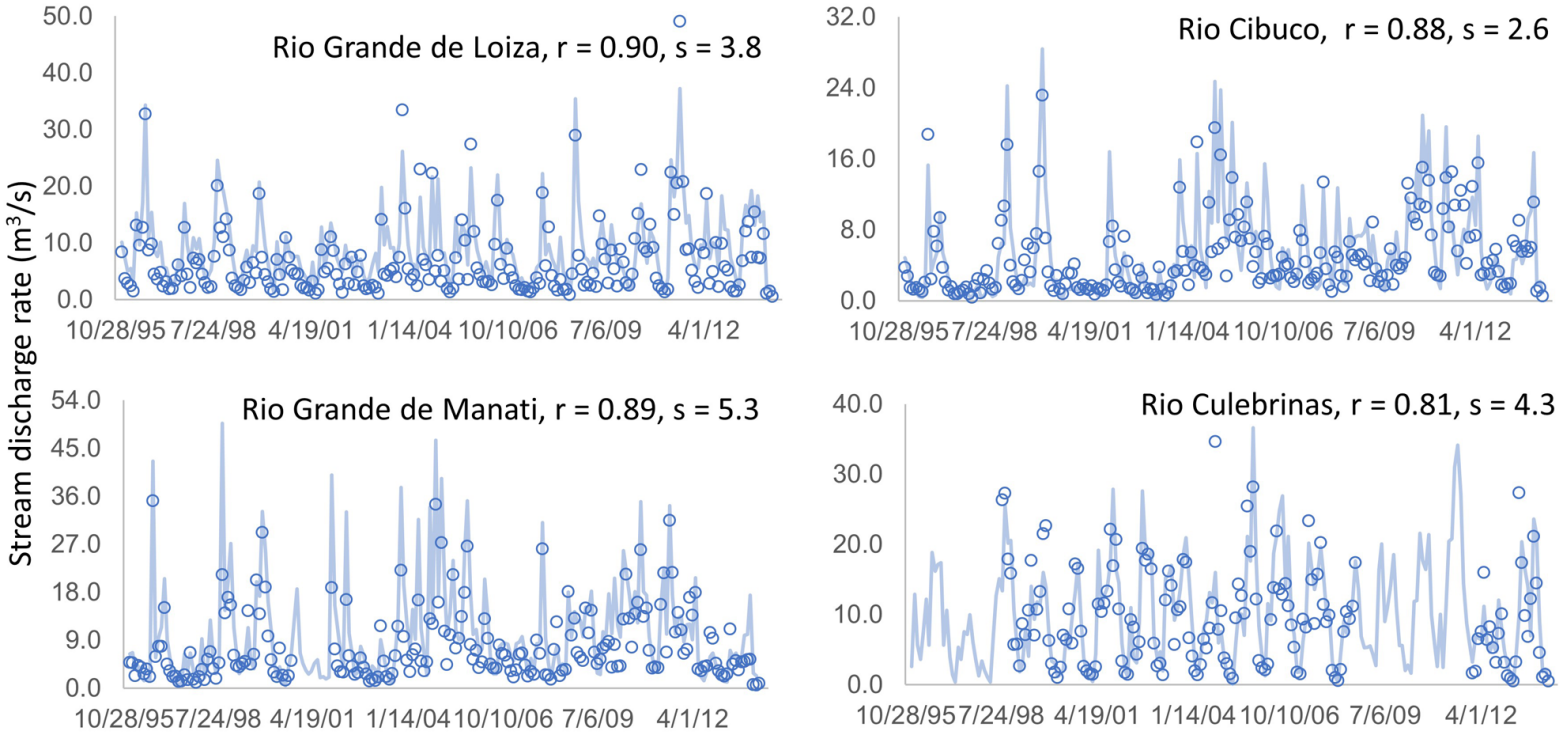
Simulated monthly mean discharge with the land cover map of 2000 versus USGS gauged data for LOZ, CBC, MNT, and CLR. All the correlation coefficients have *p*-values less than 0.01. *r* is the Pearson’s correlation coefficients, *s* is the root mean square of the error.

The simulated monthly average discharge rate in m^3^ s^-1^ using the land cover/use map of 2000 was compared to the corresponding gauged discharge during 1996–2013 (Figs 2 and 3) to assess the simulation quality. Pearson’s correlation coefficients between the observed and the simulated discharges ranged from 0.81 to 0.94, and root mean square of error varied from 0.5 to 5.3. The error increased with the amplitude of temporal variation (maximum discharge), and may be caused by the missing rainfall and temperature sequences and the unknown water use from the aquifer by local people. The unknown water use from the two mountain reservoirs may be responsible for the big error in the Rio Grande de Manati watershed.

### Land cover changes of the eight watersheds

All watersheds except Rio Blanco had forest cover increased in 1977–1991 (Table 2). Except Rio Cibuco watershed which kept the reforestation, most watersheds experienced slight deforestation or stable forest cover from 1991 to 2000. The Rio Culebrinas watershed had the strongest reforestation with forest cover changing from 7% in 1977 to 41% in 1991. The Rio Blanco and the Rio Espiritu Santo watersheds maintained 90% forest cover due to the protection of the El Yunque National Forest. The average forest cover of all watersheds increased from 45% in 1977 to 58% in 1991 and then slightly decreased to 56% in 2000. Excluding the two protected watersheds (Rio Blanco and Rio Espiritu Santo), we found the average forest cover increased from 30% in 1977 to 46% in 1991, indicating more than 50% relative increase. Perimeter-to-area ratio was only computed for the maps of 1991 and 2000 (Table 2) due to the incompatible methodology used to derive the map in 1977. The forest perimeter-to-area ratio differed greatly among watersheds from 0.004 for Rio Espiritu Santo and Rio Blanco with 90% forest cover, to 0.03 m^-1^ for Rio Cibuco in 1991 and Rio Culebrinas in 2000. The perimeter-to-area ratio of forest in all watersheds maintained or increased from 1991 to 2000 except Rio Cibuco which was the only watershed keeping reforestation trend and thus had a decreased forest perimeter-to-area ratio.

**Table 2:** Landscape parameters and annual rainfall averaged over the 1981–2013 period for eight watersheds. *A* stands for land cover fraction of total watershed area, *P* is perimeter-to-area ratio, an index for shape irregularity and patchiness or fragmentation. Subscripts *a*, *u*, *f*, and *g* stand for agriculture, urban land, forest, and grassland/pasture, respectively.

WTSD	Year	*A*_*a*_ (%)	*P*_*a*_ (m^-1^)	*A*_*u*_ (%)	*P*_*u*_ (m^-1^)	*A*_*f*_ (%)	*P*_*f*_ (m^-1^)	*A*_*g*_ (%)	*P*_*g*_ (m^-1^)	Rain (m)
FJD	1977	2.0		1.1		61.3		35.6		2.55
	1991	0.0	0.111	8.3	0.060	68.3	0.011	23.4	0.036	
	2000	0.2	0.107	5.1	0.071	64.5	0.011	30.2	0.029	
ESS	1977	0.0		3.0		89.0		8.0		3.74
	1991	0.0	0.000	3.3	0.053	94.2	0.004	2.5	0.073	
	2000	0.1	0.112	3.3	0.056	93.0	0.004	3.6	0.066	
BLC	1977	0.2		2.5		90.2		7.0		3.44
	1991	0.0	0.125	2.4	0.056	90.1	0.004	7.4	0.046	
	2000	0.2	0.110	2.7	0.058	90.1	0.004	7.0	0.048	
GRB	1977	6.0		9.5		16.0		68.5		1.77
	1991	6.4	0.005	18.3	0.051	34.1	0.028	41.1	0.034	
	2000	1.4	0.088	17.9	0.046	25.3	0.028	55.4	0.024	
LOZ	1977	4.4		8.7		25.6		61.3		2.12
	1991	0.5	0.105	14.4	0.060	48.1	0.026	37.0	0.041	
	2000	1.7	0.098	14.9	0.057	43.5	0.027	39.8	0.040	
CBC	1977	11.0		9.7		34.4		44.9		1.85
	1991	0.9	0.065	13.0	0.056	36.8	0.030	49.2	0.030	
	2000	0.9	0.100	15.1	0.053	44.9	0.025	39.2	0.038	
MNT	1977	23.6		3.6		37.9		34.8		2.04
	1991	12.1	0.064	9.0	0.074	49.9	0.022	29.0	0.043	
	2000	12.4	0.069	10.2	0.071	50.0	0.023	27.4	0.046	
CLR	1977	67.1		8.8		6.9		17.2		2.29
	1991	2.3	0.032	15.2	0.060	41.3	0.026	41.2	0.036	
	2000	2.8	0.079	15.8	0.058	37.5	0.030	43.9	0.036	

Except the two watersheds in the El Yunque National Forest, the mountainous watersheds have relatively large pasture cover. The average grassland/pasture cover of the six watersheds outside the National Forest decreased from 44% in 1977 to 37% in 1991 and then increased to 39% in 2000. Decrease in pasture cover from 1977 to 1991 was associated with increase in forest cover for all the watersheds except Rio Culebrinas and Rio Cibuco for which pasture cover increased from the abandoned agriculture (Table 2). Pasture cover increased slightly from 1991 to 2000 for all the watersheds except Rio Cibuco and Rio Grande de Manati, and the increased pasture cover was associated with decreased forest cover. The average pasture perimeter-to-area ratio across all watersheds decreased slightly from 0.042 in 1991 to 0.04 in 2000. The increased pasture covers were all associated with reduced perimeter-to-area ratios, while decreased covers with enhanced perimeter-to-area ratios.

Most of the mountainous watersheds had agriculture cover less than 12% except Rio Culebrinas and Rio Grande de Manati, which had 67% and 24% agriculture in 1977 respectively. The agricultural cover of Rio Culebrinas decreased to about 3% and that of Rio Grande de Manati to 12%, giving rise to the forest, pasture, or urban development. However, there was a slight increase in agriculture during 1991–2000 for all the watersheds except Rio Gurabo and Rio Cibuco.

**Fig 4:**
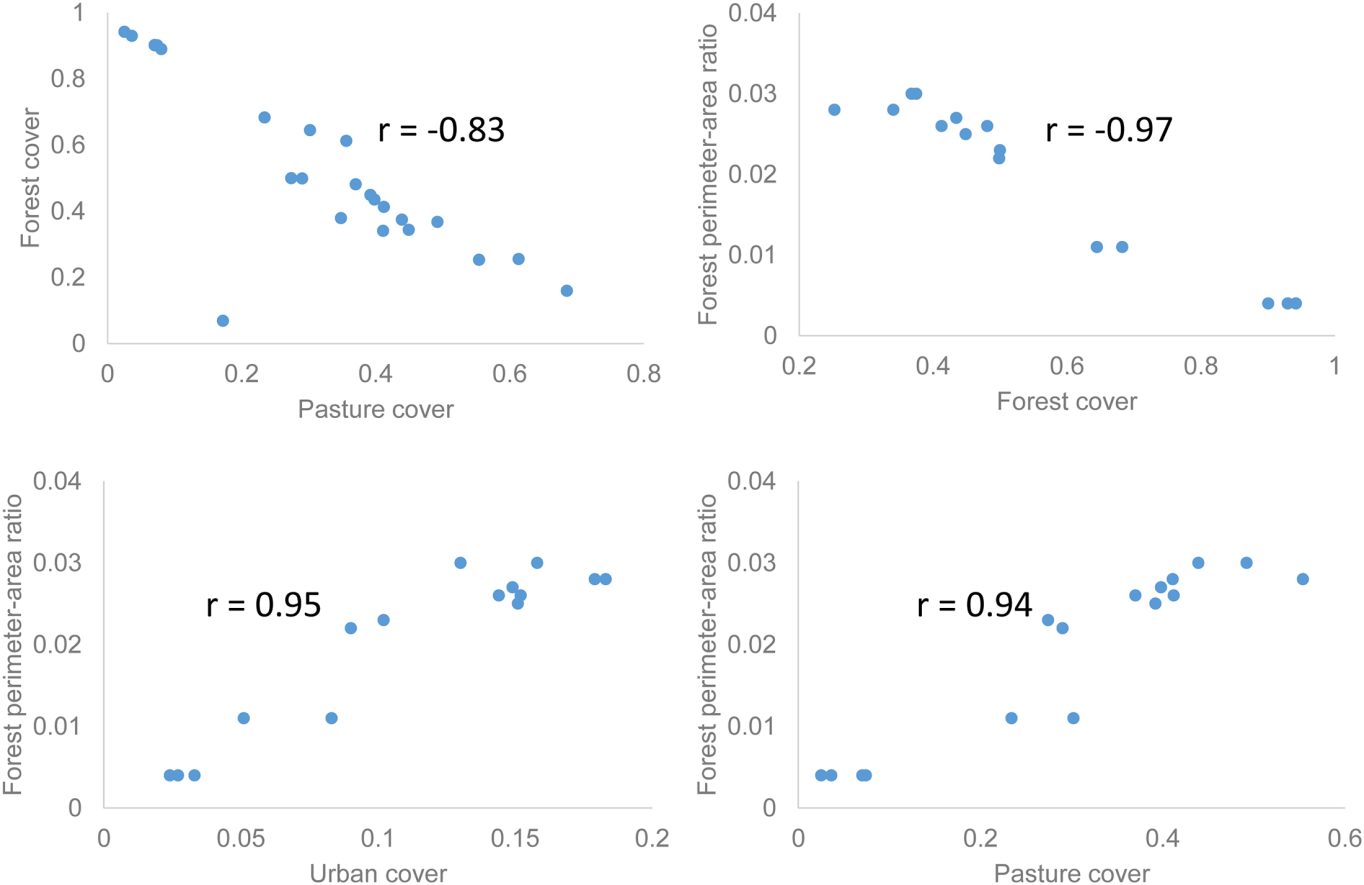
Correlations among land covers and perimeter-to-area ratio of forest.

We found strong correlations among the land cover types (Fig 4). In particular, the Pearson’s correlation between forest cover and pasture cover across all watersheds was -0.83, and that between forest cover and agricultural cover was -0.50, indicating the reforestation was mostly from pasture and abandoned agriculture.

Fragmentation was negatively correlated to the cover of the land cover type itself, but may be positively correlated with the cover of other land cover types (Fig 4). We found the forest perimeter-to-area ratio had a correlation coefficient of -0.97 with forest cover, and the pasture cover and its perimeter-to-area ratio had a correlation coefficient of -0.84. Therefore, reforestation reduced forest fragmentation. Forest perimeter-to-area ratio was positively correlated with covers of pasture and urban land, and the correlation coefficients were 0.94 and 0.95, respectively (Fig 4). Hence the fragmentation of forest may be caused by deforestation for pasture and urban expansion. Compared to forest, the perimeter-to-area ratio of pasture was positively correlated to forest cover with coefficient of 0.77, hence forest regrowth may increase the fragmentation of pasture. The perimeter-to-area ratio of pasture and that of forest also significantly correlated with a coefficient of -0.66. The abovementioned correlation coefficients were all significant at *p*<0.05 level.

### Analyses of long-term averaged discharge and big steam flow as functions of land cover/use composition and configuration

For the simulations using the land cover/use maps of 1977 and 1991, we found the average annual discharge per watershed area *F* (Mt y^-1^ km^-2^) was significantly increased by the average annual rainfall, *R* (m), but reduced by the forest cover, *C*_*f*_ (Eq 1).

$$F_{77-91}=-0.87+1.03R-0.30C_{f}$$ (1)

Average discharge in the mountainous tropical watersheds was primarily controlled by rainfall variation which explained 91% of the total sum of regression squares. Changes in forest cover only explained 9%. The big stream flow per watershed area (*F*_*b*_) was also increased by the rainfall but decreased by the cover of forest (Eq 2).

$$F_{b,77-91}=0.33R-0.52C_{f}$$ (2)

In contrast to the average discharge, the variation of forest cover explained 51% of the sum of regression squares, while the effects of rainfall largely decreased. When the two watersheds within the National Forest were excluded from the regressions, we found the coefficients were slightly altered (Eqs 3 and 4).

$$F_{77-91}=-0.91+1.05R-0.31C_{f}$$ (3)

$$F_{b,77\_91}=0.32R-0.52C_{f}$$ (4)

However, the forest cover explained 62% and 61% for the average and the big discharges, respectively.

For the simulations with the land cover/use maps of 1991 and 2000, the average stream discharge per watershed area was found as a function of rainfall and perimeter-to-area ratio of grassland/pasture (*P*_*g*_) (Eq 5).

$$F_{91-00}=-0.81+0.97R-1.99P_{g}$$ (5)

The average discharge was increased by rainfall, but reduced by the perimeter-to-area ratio of pasture which explained only 1% of the total sum of squares of the fixed effect model. The big flow per watershed area was found to be reduced by increased forest cover and its perimeter-to-area ratio (Eq 6), and the two variables accounted for 13% and 3% of the total sum of squares, respectively.

$$F_{b,91-00}=0.31R-0.37C_{f}-3.24P_{f}$$ (6)

For the maps of 1991 and 2000, we also redid the regressions with exclusion of the two watersheds within the National Forest, and found different relationships (Eqs 7 and 8).

$$F_{91\_00}=-0.53+0.87R-0.31C_{f}-1.59P_{f}$$ (7)

$$F_{b,91-00}=0.68-0.37C_{f}-3.46P_{f}$$ (8)

The big discharge was not significantly affected by rainfall. Both the average and the big discharges were significantly decreased with forest cover and its perimeter-to-area ratio. Forest cover and its perimeter-to-area ratio explained 86% and 4% of the sum of squares for the average discharge, and 82% and 18% of that for the big discharge, respectively. All the coefficients in Eq (1) through Eq (8) had one-tailed *p*-values less than 0.05 according to the LME.

## Discussion

The reforestation from abandoned agriculture and pasture in Puerto Rico is an important feature of the mountainous watersheds from 1977 to 1991. Increased forest cover in general reduces forest fragmentation. This conclusion is validated by the results at both individual watershed and multi-watersheds scales, and conforms to early findings [42]. In contrast to reforestation, deforestation due to urban sprawl and reclamation for agriculture increases forest fragmentation [11, 22]. Human activities driven by the changes in policy and economy played the primary role in this transition [20, 43].

Land cover changes largely impact stream discharges of the tropical mountainous watersheds. In 1970s-1990s, reforestation prevailed and forest cover explained around 60% of the total variation in the average and the big discharges per watershed area when the Rio Espiritu Santo and the Rio Blanco under national forest protection were excluded. When the two protected watersheds were included, the forest cover still explained 51% of the total sum of squares of the big discharges per watershed area. Our finding is comparable to previous studies [44] which concluded that landscape composition and configuration explained 64% of freshwater supply. Hence during the twenty years of fast reforestation, the increases in forest cover significantly reduced the stream discharge and the freshwater supply. On the other hand, the enhancement of forest cover also substantially reduced the big stream discharges, thus reduced the risk of flooding and landslides. The absence of the changes in the land cover type other than forest in the regression equation might be attributed to the prevailing signal of reforestation in this period. Land fragmentation indices were not applied in these regressions due to the incompatible resolution of the land cover map in 1977.

During late 90s to the new century, reforestation in Puerto Rico slowed down. The perimeter-to-area ratio of pasture appeared in the regression for average discharges despite its small contribution to the fixed component of the dependent variable (Eq 5). In the regression for big discharge and the regressions of average and big discharges without the two protected watersheds, forest cover accounted for up to 86%, and forest perimeter-to-area ratio for up to 18%, of the total variation of the dependent variables in the fixed models, in contrast to the previous conclusion that landscape configuration did not contribute significantly to the freshwater supply [44]. Decrease in forest cover during 1991–2000 significantly increased both average and big discharges for most watersheds. However, increase in forest fragmentation associated with the deforestation significantly reduced average and big discharges. The reason why forest cover did not appear in Eq 5 may have something to do with the great and stable forest cover of Rio Espiritu Santo and Rio Blanco, which may obscure signals of the changes in forest cover of other watersheds. Changes in forest cover and forest fragmentation have stronger impacts on big discharges than on average discharges, as shown by the greater percentages of explained variations in the regressions (Eqs 1–8). Forest has deeper root system and greater water budget than other land cover types, thus can buffer the extreme rainfall events and smoothen the stream flows. Our finding agrees with those found in the literature that reforestation reduces, but deforestation increases, the average discharge in the tropical watersheds [45, 46].

Current literature showed equivocal consequence of changes in land configuration on hydrological service. Deforestation and forest fragmentation were found to increase hydrological connectivity [31] in the forest dominated landscape. On the other hand, forest fragmentation exposes more edges to solar radiation, so that water loss via transpiration is enhanced with fragmentation. More importantly, fragmented forest with more edges may have greater chances to intercept the overland and subsurface lateral flows along topographical slopes, so that forest can have more water to evaporate, than an aggregated forest. Hence the fragmented forest may also reduce the average stream discharges and the big flows. Our finding contradicts the argument that landscape fragmentation facilitates the delivery of ecosystem service [25, 47].

Forest perimeter-to-area ratio is negatively correlated with forest cover. Reforestation is associated with reduced fragmentation, but deforestation is associated with increased forest fragmentation. When increased forest cover tends to reduce the stream discharges, the decreased forest fragmentation tends to enhance the stream discharges. On the other hand, when forest cover decreases during deforestation, the increased forest fragmentation tends to reduce the stream discharges. Thus changes in forestation fragmentation tend to offset the impacts of changes in forest cover in terms of regulation of freshwater supply and flooding risks.

The island of Puerto Rico has been under economic crisis in the past decade and the government decided to foster its agriculture sector. The recently issued land use plan calls for reserving at least 235,527 ha (582,000 acres) land with agriculture value which is mostly located in the central mountains (http://www.jp.pr.gov/). The land cover map of 2000 has already shown the deforestation for agriculture and pasture [20] despite the main trend of reforestation. The deforestation in the central mountains may continue to decrease the forest cover, but increase forest fragmentation. The net effect of these changes may increase the amount of freshwater supply, but on the other hand, raise the large discharges during episodic storm events.
